# Antimicrobial Resistance Genes, Cassettes, and Plasmids Present in *Salmonella enterica* Associated With United States Food Animals

**DOI:** 10.3389/fmicb.2019.00832

**Published:** 2019-04-17

**Authors:** Elizabeth A. McMillan, Sushim K. Gupta, Laura E. Williams, Thomas Jové, Lari M. Hiott, Tiffanie A. Woodley, John B. Barrett, Charlene R. Jackson, Jamie L. Wasilenko, Mustafa Simmons, Glenn E. Tillman, Michael McClelland, Jonathan G. Frye

**Affiliations:** ^1^Department of Microbiology, University of Georgia, Athens, GA, United States; ^2^Bacterial Epidemiology and Antimicrobial Resistance Research Unit, United States Department of Agriculture, Agricultural Research Service, Athens, GA, United States; ^3^Department of Biology, Providence College, Providence, RI, United States; ^4^INSERM, CHU Limoges, RESINFIT, University of Limoges, Limoges, France; ^5^Eastern Lab, United States Department of Agriculture, Food Safety and Inspection Service, Athens, GA, United States; ^6^Department of Microbiology & Molecular Genetics, University of California, Irvine, Irvine, CA, United States

**Keywords:** *Salmonella*, plasmids, antimicrobial resistance, agriculture, integrons

## Abstract

The ability of antimicrobial resistance (AR) to transfer, on mobile genetic elements (MGEs) between bacteria, can cause the rapid establishment of multidrug resistance (MDR) in bacteria from animals, thus creating a foodborne risk to human health. To investigate MDR and its association with plasmids in *Salmonella enterica*, whole genome sequence (WGS) analysis was performed on 193 *S. enterica* isolated from sources associated with United States food animals between 1998 and 2011; 119 were resistant to at least one antibiotic tested. Isolates represented 86 serotypes and variants, as well as diverse phenotypic resistance profiles. A total of 923 AR genes and 212 plasmids were identified among the 193 strains. Every isolate contained at least one AR gene. At least one plasmid was detected in 157 isolates. Genes were identified for resistance to aminoglycosides (*n* = 472), β-lactams (*n* = 84), tetracyclines (*n* = 171), sulfonamides (*n* = 91), phenicols (*n* = 42), trimethoprim (*n* = 8), macrolides (*n* = 5), fosfomycin (*n* = 48), and rifampicin (*n* = 2). Plasmid replicon types detected in the isolates were A/C (*n* = 32), ColE (*n* = 76), F (*n* = 43), HI1 (*n* = 4), HI2 (*n* = 20), I1 (*n* = 62), N (*n* = 4), Q (*n* = 7), and X (*n* = 35). Phenotypic resistance correlated with the AR genes identified in 95.4% of cases. Most AR genes were located on plasmids, with many plasmids harboring multiple AR genes. Six antibiotic resistance cassette structures (ARCs) and one pseudo-cassette were identified. ARCs contained between one and five resistance genes (ARC1: *sul*2, *strAB, tetAR*; ARC2: *aac3-iid*; ARC3: *aph, sph*; ARC4: *cmy-2*; ARC5: *floR*; ARC6: *tetB*; pseudo-ARC: *aadA, aac3-VIa, sul*1). These ARCs were present in multiple isolates and on plasmids of multiple replicon types. To determine the current distribution and frequency of these ARCs, the public NCBI database was analyzed, including WGS data on isolates collected by the USDA Food Safety and Inspection Service (FSIS) from 2014 to 2018. ARC1, ARC4, and ARC5 were significantly associated with cattle isolates, while ARC6 was significantly associated with chicken isolates. This study revealed that a diverse group of plasmids, carrying AR genes, are responsible for the phenotypic resistance seen in *Salmonella* isolated from United States food animals. It was also determined that many plasmids carry similar ARCs.

## Introduction

Non-typhoidal *Salmonella enterica* is one of the most common causes of foodborne illnesses globally, with an estimated 1.2 million cases each year in the United States alone (CDC, 2013). Symptoms range from self-limiting gastrointestinal illness to sepsis. These infections can lead to death unless treated with antibiotics (Crump et al., 2015). Unfortunately, antimicrobial resistance (AR) has been increasing since the 1980s (Crump et al., 2015). The Center for Disease Control and Prevention (CDC) considers drug-resistant non-typhoidal *Salmonella* to be a serious level threat to human health, and currently reports that 8% of *Salmonella* infections are either multidrug resistant (resistant to three or more classes of antimicrobials), or resistant to an antibiotic used for treatment, such as ceftriaxone and ciprofloxacin (CDC, 2013).

Up to 94% of United States *Salmonella* infections are estimated to be foodborne, demonstrating the importance of investigating *Salmonella* isolated from food animals (Scallan et al., 2011). The National Antimicrobial Resistance Monitoring System (NARMS) tracks antimicrobial susceptibility of bacteria associated with animals, retail meat, and foodborne illness in humans. In 2015, 21.3% of animals tested by NARMS were positive for *Salmonella* with individual sources as low as 8% in beef cattle and as high as 50% in sows, based on cecal sampling. Retail meat isolates in 2015 were positive for *Salmonella* at a lower percentage in all sources (4.3%). Individual sources ranged from 0.4% (ground beef) to 6.2% (ground chicken). Of the *Salmonella* isolated by NARMS, 35.3% of the animal samples, and 57.7% of the retail meat samples, were resistant to at least one antibiotic (FDA, 2015).

For many *Salmonella*, AR genes are carried on a mobile genetic element (MGE) (Carattoli, 2003). MGEs, like plasmids, have been shown to be extremely important in the expansion of AR genes in *Salmonella* and other Enterobacteriaceae, such as *Klebsiella pneumoniae* and *Escherichia coli* (Carattoli, 2013; Gillings, 2014). Plasmids specifically have been identified carrying AR genes in hospital-acquired infections, community-acquired outbreaks, and have also been associated with AR genes in isolates from animals raised for consumption (Conlan et al., 2016; Folster et al., 2017; Tate et al., 2017).

*Salmonella* are capable of harboring multiple, large, conjugative plasmids that can carry AR genes encoding resistance to several classes of antibiotics, including β-lactams, tetracyclines, aminoglycosides, and quinolones (Johnson et al., 2010; Glenn et al., 2011; Jain et al., 2018). However, while one cell can harbor multiple plasmids, they must be of different incompatibility groups. Plasmids of the same incompatibility group are unlikely to persist in the same isolate, while plasmids of different groups can usually coexist without issue (Novick, 1987). Incompatibility can be predicted by typing plasmids based on the replicon-associated genes they contain (Carattoli et al., 2005). Plasmids of several different incompatibility groups have been associated with multiple AR genes in *Salmonella* and other bacteria (Carattoli, 2009). For example, IncA/C plasmids isolated from *Salmonella* have been associated with genes conferring resistance to aminoglycosides, β-lactams, chloramphenicol, sulfisoxazole, tetracyclines, and trimethoprim (Hoffmann et al., 2017). Recently analyzed human infection isolates from the 1960s implicate F, I1, X1, and N type plasmids as early carriers of β-lactam resistance genes in *Salmonella* (Tran-Dien et al., 2018).

Integrons have also been shown to be important to the spread of AR in both clinical and agricultural isolates of *Salmonella* (Kaushik et al., 2018). Integrons have a well-defined structure consisting of: an integrase gene, which catalyzes the integration of new genes, the *att*I recombination site where the new genes integrate, and a promoter to express incorporated genes. The incorporated genes are called gene cassettes and are often AR genes (Gillings, 2014). The arrangement of these genes is used to assign them numbers based on the Integrall database of known integron sequences (Moura et al., 2009). While not independently mobile, integrons can be mobilized by other elements, like plasmids or transposons (Partridge et al., 2018).

Despite the established link between plasmids and AR genes, there is less known about the prevalence and characteristics of plasmids containing AR genes in isolates from food animals (Carattoli, 2003). Considering the high incidence of foodborne infection in the United States, and increasing AR, understanding the complete picture of AR in *Salmonella* is crucial. To investigate this relationship, 193 animal-associated *S. enterica* isolates of diverse serotypes and phenotypic resistance profiles, collected by NARMS from 1998 to 2011, were selected for this study. Whole genome sequence analysis (WGS) identified plasmids, AR genes, integrons, and AR cassettes (ARCs) present in these isolates. To determine the current relevance of these ARCs, publicly available genomic data of *S. enterica* from food animals collected by the USDA Food Safety and Inspection Service (FSIS) from 2014 to 2018 (*n* = 6681), were analyzed for the presence of the ARCs. Their association with plasmid replicons was determined. This is the first WGS analysis of isolates from the NARMS animal collection, which represent the first 15 years of this United States program. Combined with analysis of WGS data from the most recent 5 years of HAACP FSIS isolates, this is the most comprehensive nationwide study of AR in *Salmonella* associated with food animals. The associations of ARCs and MGEs identified in this study improve our understanding of AR in United States food animals, and may help us predict and prevent further spread of AR in *Salmonella*.

## Materials and Methods

### Isolates

One hundred and eighty nine *S. enterica* isolates, with collection dates ranging from 1998 to 2011, were selected from the NARMS animal isolate collection for the retrospective part of this study (Gupta et al., 2016a,b,c,d,e,f,g,h; Karp et al., 2017). In addition, four serotype Heidelberg isolates from a 2011 outbreak in humans were selected from the California Department of Health (Hoffmann et al., 2012). To maximize the AR gene diversity of the *Salmonella* in the retrospective study, isolates were selected based on differences in phenotypic AR profile, serotype, and the uncommon nature of their Pulsed-Field Gel Electrophoresis (PFGE) patterns within the PulseNet database. Eighty-six different serotypes and serotype variants were represented in this isolate set. These bacteria were isolated from various animal and animal associated sources, such as carcass rinses and swabs, ground product, the processing environment, sick animals, and infected humans. Animal associated sources included poultry, swine, cattle, horses, wild reptiles, wild mammals, companion animals, and associated processing environments (Supplementary Table S1). All patient information was blinded for the human isolates to insure confidentiality.

Additionally, WGS data of *S. enterica* isolates recently collected for Hazard Analysis and Critical Control Points (HACCP) verification testing by USDA-FSIS from chicken, turkey, pork, or beef products, were evaluated. Isolation procedures are described in the USDA-FSIS Microbiology Laboratory Guidebook (MLG) Chapter 4 (Dey and Lattuada, 1998). Only WGS data was used from these isolates as phenotypic data was not available. Isolates were selected based on publicly available data in NCBI’s Pathogen Detection Isolate Browser^1^.

### Phenotypic Antimicrobial Susceptibility Testing

For the 193 retrospective isolates collected from 1998 to 2011, phenotypic susceptibility to 14 different antibiotics (Supplementary Table S1) was determined by broth-microdilution. The Sensititre semi-automated antimicrobial susceptibility system (TREK Diagnostic Systems Inc., Cleveland, OH, United States) was used to inoculate the Sensititre custom NARMS plate CMV3AGNF per manufacturer’s instruction. The minimum inhibitory concentration (MIC) and classification as resistant, susceptible, or intermediate for each of the 14 antibiotics were assigned using breakpoints set by the Clinical and Laboratory Standards Institute (CLSI, 2016). For antibiotics without CLSI established breakpoints, NARMS breakpoints were used^2^.

### Genome Sequencing, Assembly, AR Gene, and Integron Identification

Total DNA was extracted using a Sigma GenElute kit (Sigma Life Sciences, St. Louis, MO, United States). Libraries were prepared according to the Illumina protocol using the Nextera XT DNA sample preparation kit. Isolates were sequenced using an Illumina HiSeq2500 (Illumina, San Diego, CA, United States) at The Genome Institute at Washington University in St. Louis, MO, United States. Reads were assembled into draft sequences using A5 with default settings, including quality trimming (Tritt et al., 2012). Draft genomes were annotated with Prokka using default settings (Seemann, 2014). All sequences had greater than 40× coverage, an average N50 of greater than 350,000, and an average of 116 contigs (median of 97 contigs) (Supplementary Table S2). AR genes were identified using ARG-ANNOT V3 (Gupta et al., 2014). Integrons were identified using Integrall (Moura et al., 2009).

Regulatory isolates collected and sequenced by the USDA-FSIS from 10/31/2014 to 4/16/2018 were also included for analysis. WGS data was generated from MiSeq libraries prepared using the Nextera XT library prep kit (Illumina, San Diego, CA, United States) and sequenced on the Illumina MiSeq platform using either 300 Cycle or 500 Cycle Version 2 chemistries. The raw files were assembled using either CLC Genomics Workbench v8 or v11 (Qiagen) or SPAdes version 3.7.0^3^ (St. Petersburg, Russia).

### Plasmid Identification

Plasmid replicon-associated genes were detected using BLASTN to identify the target sequence in the genomes of each isolate (Camacho et al., 2009). Target sequences were selected based on plasmid replicon typing as well as relaxase typing schemes (Carattoli et al., 2005; Villa et al., 2010; Compain et al., 2014). Additional contigs belonging to plasmids not identified in the replicon and relaxase BLAST were identified using BLASTN against a custom plasmid BLAST database. The custom database was created by extracting all plasmids from NCBI that were associated with Enterobacteriaceae as of March 2015 (Coordinators, 2017). The additional plasmid contigs were confirmed using the following criteria: First, contigs that were identified in the replicon/relaxase BLAST were used to identify the primary reference plasmid, meaning, the plasmid in the custom database that aligned to the initial BLAST identified contig with the greatest coverage and percent identity. Second, large contigs (>10,000 bp) not identified in the initial BLAST that aligned with high identity (>70%) and coverage (>40%) to the primary reference plasmid for a specific replicon and did not have substantial homology with another replicon were binned as part of the same plasmid. For these large contigs, a 70% cut-off for identity was chosen based on the range of percent identities of the primary reference plasmids to the contigs containing the replicon or relaxase genes. A 40% cut-off for coverage was chosen to allow for contigs that were continuous where the reference sequence was not, i.e., for cases where the reference plasmid and the contig being queried began in different places and there was a large gap between homologous sequences resulting in two different BLAST hits for the identified contig. Third, smaller contigs (3,000–10,000 bp), that aligned to reference plasmids, different than the primary, but of the same replicon type, and those with lower identity were also binned if they matched the reference plasmid or a plasmid of the same replicon type. Contigs binned together were extracted and used to create a plasmid draft. Contigs were included in drafts only if they could not be associated with another plasmid of a different replicon type. Single contigs that aligned with an entire plasmid in the BLAST analysis, but were not identified in the initial BLAST, were considered separate plasmids. ColE replicons were not processed into draft sequences due to the short length of contigs and difficulty in assembly. However, contigs that contained both a ColE replicon and an AR gene were analyzed. Drafts were annotated with RAST (Overbeek et al., 2014). Replicon types with an established Plasmid Multi Locus Sequence Type (pMLST) scheme were typed by querying the pMLST database^4^ (Garcia-Fernandez et al., 2008, 2011; Garcia-Fernandez and Carattoli, 2010; Jolley and Maiden, 2010; Hancock et al., 2017). Contig coverage was also analyzed for each sequence using Bowtie2 and Qualimap (Garcia-Alcalde et al., 2012; Langmead and Salzberg, 2012).

### Antibiotic Resistance Cassette Identification

For AR genes that were identified in multiple retrospective isolates, the contig containing the gene was aligned with the contigs containing the gene from other isolates using SnapGene^5^. Homologous sequence among these isolates immediately adjacent to the resistance gene was considered an Antibiotic Resistance Cassette (ARC). ARC sequences were defined as the sequence including an identical AR gene with identical flanking sequence, allowing for up to five base pair mismatches, in multiple unrelated isolates. ARC sequences were compared to retrospective isolates containing the AR gene, but not the entire ARC, using BLASTN to identify additional isolates containing the ARC sequence split onto multiple contigs (Camacho et al., 2009). ARC sequences were compared to the NCBI non-redundant (nr) database using BLASTN to identify matching sequences, and to identify the species and prevalence of sequenced isolates containing these ARC sequences.

Antibiotic resistance cassette sequences were also compared to the USDA-FSIS *Salmonella* isolates, using BLASTN. Isolates were only included in the comparison if they were predicted to contain the ARC. Predictions were based on the presence of the ARC AR genes in each isolate as presented by the Pathogen Detection Isolate Browser. Isolates were considered to contain the ARC if the whole ARC sequence was present or if the sequence was overlapping on multiple contigs.

### Statistics

Ratio of FSIS isolates containing ARCs (animal source and serotype) were compared using 95% confidence intervals (95% CI) calculated in R. Conditional probabilities were calculated in Excel for isolates containing multiple ARCs using the following formulas:

$$\begin{array}{c} P\left(A|B\right)=\frac{P\left(A\;and\;B\right)}{P\left(A\right)}\;\\ P\left(A|B|C\right)=\frac{P\left(A\;and\;B\;and\;C\right)}{P\left(A\;and\;B\right)*P\left(A\right)} \end{array}$$

## Results

### Phenotypic and Genotypic Antimicrobial Resistance

The retrospective study utilized WGS to analyze 193 isolates collected from 1998 to 2011. Phenotypic AR was known prior to sequencing and was used to help select the isolates for this study. Selected isolates (*n* = 119) exhibited phenotypic resistance to at least one antimicrobial tested and 67 of those were multi-drug resistant (resistant to three or more classes of antimicrobial). Resistance was observed for 13 of 14 antimicrobials tested in at least one isolate, with no resistance seen to ciprofloxacin. The most common ARs in the data set were to tetracycline, streptomycin, ampicillin, and sulfamethoxazole or sulfisoxazole (Supplementary Table S1).

A total of 923 AR genes were identified from the sequences (Table 1). All 193 retrospective isolates contained at least one AR gene (Supplementary Table S1). The most frequently identified AR gene was *aac*(6′)-I, an aminoglycoside acetyltransferase gene, variants *aac*(6′)-I-y (*n* = 159) and *aac*(6′)-I-aa (*n* = 30) that was present in almost every isolate. Setting aside *aac*(6′)-I, other genes for resistance to aminoglycosides were still the most numerous followed by genes for resistance to tetracyclines and β-lactams (Table 1). AR gene presence corresponded with phenotypic AR for 95.4% (618/648) of genes for which phenotypic testing was completed (Supplementary Table 1). One hundred and twenty six isolates were considered MDR as they contained multiple AR genes for multiple classes of antimicrobials (Supplementary Table S1).

**Table 1:** Resistance genes identified and associated with plasmids in the retrospective isolates (*n* = 193).

Antibiotic resistance gene	Antibiotic Class to which resistance is conferred	Number of genes identified in *n* = 193 isolates	Predicted resistance conferred^∗^	Number of genes associated with plasmids
*aac*(6′)-I	Aminoglycosides	189	Gen	0
*str*B	Aminoglycosides	67	Str	57
*str*A	Aminoglycosides	65	Str	56
*aad*A (*ant*(3″)Ia)	Aminoglycosides	54	Str	38
*aac*3-Via	Aminoglycosides	22	Gen	20
*aph*(3′)Ib	Aminoglycosides	22	Kan	16
*aph*A (*aph*(3′)IIa)	Aminoglycosides	13	Kan	13
*sph* (*aph*(6)Ic)	Aminoglycosides	13	Str	13
*aac*3-Iid	Aminoglycosides	11	Gen	11
*aad*B (*ant*(2″)Ia)	Aminoglycosides	7	Gen	5
*aac-*IVa	Aminoglycosides	4	Gen	2
*aph*(4)Ia	Aminoglycosides	4	(Hygromycin)	1
*ant*(3″)Ia	Aminoglycosides	1	Str	0
*bla*_CMY -2_	β-lactams	44	Amp, Fox, Axo Amo, Tio	44
*bla*_TEM-1_	β-lactams	37	Amp	27
*bla*_CARB-3_	β-lactams	3	Amp	0
*tet*A	Tetracyclines	61	Tet	49
*tet*R^∗∗^	Tetracyclines	64	Tet	50
*tet*B	Tetracyclines	35	Tet	30
*tet*C	Tetracyclines	7	Tet	7
*tet*G	Tetracyclines	2	Tet	7
*tet*M	Tetracyclines	2	Tet	0
*sul*1	Sulfonamides	48	Sul	41
*sul*2	Sulfonamides	41	Sul	36
*sul*3	Sulfonamides	2	Sul	0
*flo*R	Phenicols	27	Chl	24
*cml*A	Phenicols	11	Chl	8
*cat*A	Phenicols	4	Chl	0
*fos*A2	Fosfomycin	48	(Fosfomycin)	0
*mph*A	Macrolides	2	Azi	2
*ere*A	Macrolides	2	(Erythromycin)	0
*mef*B	Macrolides	1	Azi	0
*dfr*A	Trimethoprim	8	Trimethoprim	5
*arr*2	Rifampicin	2	(Rifampicin)	0

*^∗^For drugs which phenotypic testing was available, only tested drugs are listed. Resistance to other drugs are possible. Drug names in parentheses were not tested. Antibiotic abbreviations are as follows: Gen, gentamicin; Kan, kanamycin; Str, streptomycin; Amp, ampicillin; Fox, cefoxitin; Axo, ceftriaxone; Amo, amoxicillin; Tio, ceftiofur; Tet, tetracycline; Sul, sulfonamide; Chl, chloramphenicol; Azi, azithromycin. ^∗∗^Indicates regulatory gene usually associated with resistance*.

### Integrons

Sixty-one isolates contained a complete integron (In). Fourteen different complete previously named integrons were identified, and six novel integrons were identified. Novel integrons were defined as an arrangement not previously sequenced and assigned a new number. In2, containing *aad*A1, was the most numerous (*n* = 21). Two isolates also contained In0, which contains no gene cassettes, but an otherwise complete integron structure. Forty-eight integrons were determined to be associated with plasmid sequences (Table 2).

**Table 2:** Number of integrons identified and integron gene cassette content in retrospective isolates (*n* = 193).

Integron	Number of isolates containing integron	Number located on plasmids	Integron gene cassettes arranged 5′–3′			
In2	21	19	*aad*A1a			
In740	9	9	*aad*A1bs			
In571	5	5	*aad*B	*gcu*E2	*gcu*8	*cml*A1g
In363	4	4	*dfr*A1	*gcu*C		
In27	2	1	*dfr*A12	*gcu*F	*aad*A2	
In0	2	0				
In142	2	2	*aad*A7			
In167	2	0	*bla*_CARB-2_			
In287	2	2	*aad*A6D2			
In839	2	1	*aad*A1bx			
In45	1	1	*aad*A12			
In127	1	0	*aad*A2			
In191	1	0	*dfr*A14b			
In862	1	1	*est*X-3	*aad*A1a		
In1581^∗^	1	0	*aad*A7g			
In1582^∗^	1	0	*dfr*A16c	*bla*_CARB-3_	*aad*A2	*ere*A1c
In1583^∗^	1	1	*aad*A31			
In1584^∗^	1	1	*est*X-6	*gcu*116		
In1585^∗^	1	0	*aad*A2	*cml*A1g	*aad*A1a	*qac*H2
In1586^∗^	1	1	*aad*A1D13			

*Integron gene cassettes are listed in order of arrangement within the DNA. gcu indicates gene cassette of unknown function (hypothetical protein). (^∗^) indicates a new integron number*.

### Plasmid Replicons Detected and Linkage to AR Genes

At least one plasmid replicon-associated gene was detected in 157 of the 193 isolates; multiple replicons were detected in 91 isolates (Supplementary Table S1). The most common types of replicon-associated genes detected were ColE followed by I1, F, X, and A/C. Additionally, HI1, HI2, Q1, and N were also detected at lower levels. A total of 212 draft plasmid sequences were created; 124 of them contained at least one AR gene and 102 contained multiple AR genes with 57 containing five or more AR genes (Tables 3–10). In total, 81.5% of AR genes were associated with a plasmid replicon (Table 1).

**Table 3:** Genotypic profiles and metadata of A/C plasmids.

Isolate	pMLST type	Serotype	Source	Plasmid genotypic
80	U	Copenhagen^∗^	C	*str*AB, *bla*_CMY -2_, *sul*2, *flo*R
169	ST1	IIIa		N/A
106	ST1	IIIa18:z4,z32:-	T	N/A
15	ST2	Copenhagen^∗^	CH	*tet*AR, *sul*1
19	ST3	Agona	S	*str*AB, *bla*_CMY -2_, *tet*AR, *sul*1, *sul*2, *flo*R, *dfr*A1
158	ST3	Agona	C	*aph*3-Ia, *str*AB, *bla*_CMY -2_, *tet*AR, *sul*1, *sul*2, *flo*R, *dfr*A1
99	ST3	Bardo	C	*str*AB, *bla*_CMY -2_, *tet*AR, *sul*2, *flo*R
75	ST3	Bredeney	T	*str*AB, *bla*_CMY -2_, *tet*AR, *sul*2
27	ST3	Dublin	C	*aph*3-Ia, *str*AB, *aad*B, *bla*_CMY -2_, *tet*AR, *sul*2, *flo*R, *cml*A
140	ST3	Dublin	C	*str*AB, *bla*_CMY -2_, *tet*AR, *sul*2, *flo*R
150	ST3	Dublin	C	*aph*3-Ia, *str*AB, *bla*_CMY -2_, *bla*_TEM-1_, *tet*AR, *flo*R
72	ST3	Give	C	*aac*3-VIa, *str*AB, *aad*A, *bla*_CMY -2_, *tet*R, *sul*1, *sul*2, *flo*R
3	ST3	Heidelberg	C	*aph*3-Ia, *str*AB, *aad*B, *bla*_CMY -2_, *tet*AR, *sul*1, *sul*2, *flo*R, *cml*A, *dfr*A1
43	ST3	Heidelberg		*str*AB, *aad*B, *bla*_CMY -2_, *bla*_TEM-1_, *tet*AR, *flo*R, *cml*A
86	ST3	Heidelberg	CH	*str*AB, *aad*B, *bla*_CMY -2_, *bla*_TEM-1_, *tet*AR, *sul*2, *flo*R, *cml*A
103	ST3	Heidelberg	T	*str*AB, *bla*_CMY -2_, *tet*AR, *sul*2, *flo*R
126, 175, 185, 187	ST3	Heidelberg	T	*str*AB, *bla*_CMY -2_, *tet*AR, *sul*2
62	ST3	IIIa 18:z4,z23:-	T	*aph*3-Ia, *str*AB, *bla*_CMY -2_, *tet*AR, *sul*1, *sul*2, *flo*R, *dfr*A1
111	ST3	Kinshasa^∗∗^	C	*str*AB, *bla*_CMY -2_, *tet*AR, *sul*1, *sul*2, *flo*R
14	ST3	Newport		*str*AB, *bla*_CMY -2_, *tet*AR, *sul*2, *flo*R
83	ST3	Newport	H	*aac*3-VIa, *str*AB, *aad*A, *bla*_CMY -2_, *tet*AR, *sul*1, *sul*2, *flo*R
139	ST3	Newport	C	*str*AB, *bla*_CMY -2_, *tet*AR, *sul*2, *flo*R
161	ST3	Ohio		*str*AB, *bla*_CMY -2_, *tet*AR, *sul*1, *sul*2, *flo*R, *dfr*A1
40	ST3	Reading	C	*aac*3-Via, *str*AB, *aad*A, *bla*_CMY -2_, *bla*_TEM-1_, *tet*A, *sul*1, *sul*2, *flo*R
17	ST3	Typhimurium	T	*aad*B, *bla*_CMY -2_, *tet*AR, *flo*R, *cml*A
125	ST3	Typhimurium	C	*str*AB, *tet*AR, *sul*2, *flo*R
132	ST3	Typhimurium	C	*str*AB, *aad*A, *bla*_CMY -2_, *tet*AR, *sul*1, *sul*2, *flo*R
39	ST3	Uganda	C	*str*AB, *bla*_CMY -2_, *tet*AR, *sul*1, *sul*2, *flo*R
50	ST3	Uganda		*str*AB, *bla*_CMY -2_, *tet*AR, *sul*1, *sul*2, *flo*R

*Isolate numbers correspond to isolate numbers with CRJJGF prefixes. Blank indicates source is unknown. U indicates unknown pMLST type due to inability to calculate. Animal source abbreviations are as follows, C, cattle; Ch, chicken; T, turkey; S, swine; H, horse. N/A indicates the plasmid contained no resistance genes. ^∗^Typhimurium variant. ^∗∗^Uganda variant*.

**Table 4:** Genotypic profiles and metadata of F plasmids.

	Replicons
Isolate	present	Serotype	Source	Plasmid genotype
46	F, FII	Braenderup	CH	*str*AB, *tet*AR
94	F, FII	Orion	CH	*aph*3″-Ia
17	F, FII, FIA, FIB	Typhimurium	T	*aph*3″-Ia, *str*AB, *tet*B, *sul*2
1, 53,	F, FII, FIB	Kentucky	PE	*str*AB, *tet*B
77, 109, 116	F, FII, FIB	Kentucky	CH	*str*AB, *tet*B
79	F, FII, FIB	Minnesota	T	*aph*3″-Ia, *str*AB, *tet*B, *sul*2
80	FIB, FIIs	Copenhagen^∗^	C	*aph*3″-Ia, *bla*_TEM-1_, *tet*AR
52	FIBs, FIIs	Choleraesuis	S	N/A
16	FIBs, FIIs	Copenhagen^∗^	C	*aph*3″-Ia, *aad*A, *bla*_TEM-1_, *tet*AR, *sul*1
5	FIBs, FIIs	Enteritidis	PE	N/A
6, 7, 8, 9, 11, 12, 13, 129, 130	FIBs, FIIs	Enteritidis	CH	N/A
10	FIBs, FIIs	Enteritidis	RTE	N/A
56	FIBs, FIIs	Enteritidis	R	N/A
131, 171	FIBs, FIIs	Enteritidis	WA	N/A
137	FIBs, FIIs	Enteritidis		N/A
29	FIBs, FIIs	I 4,[5],12:i:-	T	N/A
148	FIBs, FIIs	Kunzendorf^∗∗∗^	S	N/A
18, 96	FIBs, FIIs	Typhimurium	CH	N/A
51	FIBs, FIIs	Typhimurium	S	N/A
35	FIC	Mbandaka		*aad*A, *tet*AR, *sul*1, *dfr*A
169	FIIs	IIIa		N/A
155	FIIs	Pullorum^∗∗∗∗^	AV	N/A
125	FIIs	Typhimurium	C	*aad*A, *bla*_TEM-1_, *sul*1
28	FIIs, X1	Dublin	CH	N/A
140, 150	FIIs, X1	Dublin	C	N/A
149	FIIs, X1	Dublin	C	*bla*_TEM-1_
164	FV	Binza^∗∗∗∗∗^	T	N/A
115	FV	II 48:d:z6^∗∗^	E	N/A

*Isolate numbers correspond to isolate numbers with CRJJGF prefixes. Animal source abbreviations are as follows, C, cattle; Ch, chicken; T, turkey; S, swine; PE, poultry environment; E, environmental food contact surface; RTE, ready to eat product; AV, avian; WA, wild animal; R, wild reptile. Blank source indicates unknown. N/A indicates no resistance genes were present. ^∗^Typhimurium variant. ^∗∗^Hagenbeck. ^∗∗∗^Cholersuis variant. ^∗∗∗∗^Gallinarum variant. ^∗∗∗∗∗^Orion*.

**Table 5:** Genotypic profiles and metadata of HI plasmids.

Isolate	HI Type	Serotype	Source	Plasmid genotype
28	HI1	Dublin	CH	*str*AB, *bla*_TEM-1_, *tet*B
149	HI1	Dublin	C	*tet*B
154	HI1	Krefeld	S	*tet*B
152	HI1	Rubislaw	E	*aph*3Ia, *str*AB, *tet*B, *sul*1, *sul*2, *cml*A, *mph*A
164	HI2	Binza^∗^	T	*tet*B
70	HI2	Bovismorbificans	S	*aac*-Iva, *aph*4-Ia
63	HI2	Brandenburg	C	*aac*3-Via, *aph*3Ia, *aad*A, *tet*B, *sul*1
75	HI2	Bredeney	T	*aad*A, *aad*B, *tet*C, *sul*1
126	HI2	Heidelberg	T	*aph*A, *sph, aph*3″Ia, *tet*B
128	HI2	Heidelberg	H	*aph*A, *sph, str*AB, *tet*B
145	HI2	Heidelberg	S	*aph*A, *sph, str*AB, *tet*B
174, 184	HI2	Heidelberg	CH	*aph*A, *sph, str*AB, *tet*B
175	HI2	Heidelberg	T	*aph*A, *sph, aph*3″Ia, *tet*B
180, 181	HI2	Heidelberg	T	*aph, sph, str*B, *tet*B
185, 187	HI2	Heidelberg	T	*aph*A, *sph, tet*B
186, 194	HI2	Heidelberg	T	*aph*A, *sph, str*AB, *tet*B
81	HI2	Livingstone	E	*str*AB
156	HI2	Ouakam	CH	*tet*B
110	HI2	Putten	S	*aac*3-Iid, *aac*-Iva,*str*AB, *aad*A, *tet*B, *sul*1
159	HI2	Putten		*aad*A, *tet*B, *sul*1

*Isolate numbers correspond to isolate numbers with CRJJGF prefixes. Animal source abbreviations are as follows, C, cattle; Ch, chicken; T, turkey; S, swine; H, horse; E, environmental food contact surface. ^∗^Orion variant*.

**Table 6:** Genotypic profiles and metadata of I1 plasmids containing resistance genes.

Isolate	pMLST Type	Serotype	Source	Plasmid genotype
15	ST12	Copenhagen^∗^	CH	*bla*_CMY -2_
74	ST12	Havana	S	*bla*_CMY -2_
102	ST12	Heidelberg	cat	*bla*_CMY -2_
178	ST12	Heidelberg	T	*bla*_CMY -2_
182	ST12	Heidelberg	CH	*bla*_CMY -2_
30	ST12	Infantis	CH	*bla*_CMY -2_
44	ST12	Johannesburg	S	*bla*_CMY -2_
154	ST12	Krefeld	S	*bla*_CMY -2_
78	ST12	Minnesota	S	*bla*_CMY -2_
37	ST12	Saintpaul	T	*bla*_CMY -2_
120	ST12	Thompson	CH	*bla*_CMY -2_
116, 109	ST12, U	Kentucky	CH	*bla*_CMY -2_
141	ST155	Worthington	S	*tet*B
152	ST20	Rubislaw	E	*bla*_CMY -2_
53	ST201	Kentucky	PE	*tet*AR
142	ST222	Albany	T	*aac*3VIa, *aad*A, *sul*1
187	ST222	Heidelberg	T	*aac*3VIa, *aad*A, *sul*1
33	ST222	Schwarzengrund		*aac*3VIa, *aad*A, *sul*1
41	ST23	Cerro	C	*bla*_CMY -2_
143	ST25	Manhattan	S	*aad*A, *sul*1
23	ST26	Hadar	E	*aad*A, *sul*1
134	ST26	Hadar	T	*aac*3VIa, *aad*A, *sul*1
174, 175, 179, 180, 183, 185	ST26	Heidelberg	CH	*aac*3VIa, *aad*A, *sul*1
192	ST26	Heidelberg	T	*aac*3IId, *str*AB, *aad*A, *bla*_TEM-1_, *tet*AR
194	ST26	Heidelberg	T	*aac*3IId, *aad*A, *tet*AR
188, 189, 190, 193, 195	ST26	Heidelberg	HU	*aac*3IId, *aad*A, *bla*_TEM-1_, *tet*AR
191	ST26	Heidelberg	T	*aac*3IId, *str*A, *aad*A, *bla*_TEM-1_, *tet*AR
29	ST26	I 4,[5],12:i:-	T	*aac*3VIa, *aad*A, *sul*1
59	ST26	IIIa 18:z4,z23:-		*aac*3VIa, *aad*A, *sul*1
87	ST26	Litchfield	CH	*aac*3VIa, *aad*A, *sul*1
126, 103	ST26, U	Heidelberg	T	*aac*3VIa, *aad*A, *sul*1
40	ST4	Reading	C	*tet*CR
85	ST4	Hartford	H	*tet*C
113	U	Anatum	T	*aac*3VIa, *aad*A, *bla*_TEM-1_, *tet*B, *sul*1
45	U	Berta	T	tem
22	U	Derby	T	*tet*AR
105	U	Minneapolis^∗∗^	T	*aac*3IId, *aad*A, *bla*_TEM-1_, *tet*AR
94	U	Orion	CH	*bla*_CMY -2_
124	U	Senftenberg	C	*aph*A, *sph, sul*1

*Isolate numbers correspond to isolate numbers with CRJJGF prefixes. U indicates unknown ST type due to inability to calculate. Animal source abbreviations are as follows, C, cattle; Ch, chicken; T, turkey; S, swine; PE, poultry environmental; E, environmental food contact surface; H, horse; HU, human. ^∗^Typhimurium variant. ^∗∗^Anatum variant*.

**Table 7:** Genotypic profiles and metadata of I1 plasmids containing no resistance genes.

Isolate	pMLST	Serotype	Source
20	ST12	Agona	RTE
21	U	Montevideo	CH
31	U	Infantis	S
52	U	Choleraesuis	S
57, 77, 127	U	Kentucky	CH
75	ST80	Bredeney	T
117	U	Fresno	R
118	U	Sandiego	R
128	U	Heidelberg	H
139	U	Newport	C

*Isolate numbers correspond to isolate numbers with CRJJGF prefixes. U indicates unknown ST type due to inability to calculate. Animal source abbreviations are as follows, C, cattle; Ch, chicken; T, turkey; S, swine; RTE, ready to eat product; H, horse; R, wild reptile*.

**Table 8:** Genotypic profiles and metadata of IncN plasmids.

Isolate	pMLST	Serotype	Source	Plasmid genotype
89	N/A	Tennessee	S	N/A
133	ST1	Montevideo	C	*tet*AR
110	ST1	Putten	S	*bla*_TEM-1_
82	ST3	Javiana		*str*AB, *bla*_TEM-1_, *sul*1, *sul*2, *cml*A, *mph*A

*Isolate numbers correspond to isolate numbers with CRJJGF prefixes. Animal source abbreviations are as follows, C, cattle; S, swine; blank, unknown*.

**Table 9:** Genotypic profiles and metadata of IncQ1 plasmids.

Isolate	Serotype	Source	Plasmid genotype
91	Alachua	S	*str*AB, *tet*AR, *sul*2
177	Derby	S	*str*AB, *tet*AR, *sul*2
148	Kunzendorf^∗^	S	*str*AB, *sul*2
65	London	S	*aph*3-Id, *str*AB, *tet*AR, *sul*2
143	Manhattan	S	*str*AB, *sul*2
48	Meleagridis	C	*str*AB, *tet*AR, *sul*2
42	Muenchen	T	*str*AB, *tet*AR, *sul*2

*Isolate numbers correspond to isolate numbers with CRJJGF prefixes. ^∗^Cholersuis variant. Animal source abbreviations are as follows, C, cattle; T, turkey; S, swine*.

**Table 10:** Genotypic profiles and metadata of IncX plasmids.

Isolate	Serotype	Source	X type	Genotype
3	Heidelberg	C	X4	*bla*_TEM-1_
24	Hadar	C	X1	*bla*_TEM-1_
27	Dublin	C	X1	*bla*_TEM-1_
129	Enteritidis	CH	X1	*bla*_TEM-1_
134	Hadar	T	X1	*bla*_TEM-1_
186	Heidelberg	T	X1	*bla*_TEM-1_
139	Newport	C	X2	*aph*3″Ia
8, 13	Enteritidis	CH	X1	
25, 43	Heidelberg		X1	
37	Saintpaul	T	X1	
40	Reading	C	X1	
45	Berta	T	X1	
65	London	S	X1	
86, 95, 176	Heidelberg	CH	X1	
98	IIIb 38:(k):z35	R	X1	
102	Heidelberg	cat	X1	
103, 191, 192, 194	Heidelberg	T	X1	
104	Minneapolis^∗^	S	X1	
165, 166	IIIb 61:–:1,5,7	C	X1	
188, 190, 193, 195	Heidelberg	HU	X1	

*Isolate numbers correspond to isolate numbers with CRJJGF prefixes. Animal source abbreviations are as follows, C, cattle; Ch, chicken; T, turkey; S, swine; HU, human; R, wild reptile. Blank indicates unknown source. ^∗^Anatum variant.*

With the exception of ColE plasmids, detection of a replicon associated gene correlated with the presence of additional plasmid sequence in 100% of cases. ColE plasmids were not further characterized because the plasmids were too small to be reliably assembled. However, AR genes were detected in a few cases on the same contig with the ColE replicon, including four ColE plasmids homologous to pSC101 that contained the *tet*C gene (Supplementary Table S3).

### A/C Replicons

A/C replicon-associated genes were detected in 32 isolates, 30 of which were associated with AR genes. Eighteen different combinations of AR genes were present among these plasmids and five of the AR gene profiles were located on multiple A/C plasmids. According to the A/C pMLST scheme 27 plasmids were type ST3; the remaining four included two ST1, one ST2, and one untypable plasmid. Plasmids were present in 16 different serotypes and isolated from five different host sources. However, 14/32 plasmids were isolated from cattle sources and 9/32 were isolated from turkey sources (Table 3). These sources represented 21% and 15% of the total isolates, respectively, (Supplementary Table S1).

### F Replicons

Forty-three isolates contained at least one F type replicon-associated gene (Table 4). Because F-type plasmids can contain multiple replicon-associated genes of different types, all contigs identified as belonging to an F-replicon plasmid were considered to belong to the same plasmid. F, FII, FIIs, FIA, FIB, FIBs, FIC, and FV replicons were identified. Fourteen of these 43 draft plasmids contained AR genes. Eight different combinations of AR genes were present among these 14 isolates; five of these plasmids that contained *str*AB and *tet*B, were found in *Salmonella* Kentucky isolates from poultry. A total of eight different combinations of replicons were identified (Table 4).

### HI Replicons

Four isolates contained a HI1 plasmid and 20 isolates contained a HI2 plasmid, all of which contained AR genes. All four HI1 plasmids were from different sources, but all carried the *tet*B resistance gene. Six HI2 plasmids belonged to one resistance gene profile containing *aph, sph, str*A, *str*B, and *tet*B, while six other HI2 plasmids had unique AR gene profiles. Based on the HI1 pMLST typing scheme, two HI1 plasmids were ST2, one was ST7, and one was untypable (due to a missing allele). By the HI2 pMLST scheme, three plasmids were ST1, four ST2, and the rest untypable due to a mutation in one of the alleles used for typing (Table 5).

### I1 Replicons

Sixty-two isolates contained an I1 replicon-associated gene, yielding 62 draft plasmid sequences. Fifty of those plasmids contained AR genes. Sixteen plasmids contained only *bla*_CMY -2_ and 15 plasmids contained only three AR genes, *aad*A, *aac*3, and *sul*1 (Table 6). On 14 of those 15 plasmids; the resistance genes were associated with the integron In2; on the remaining plasmid, the genes were associated with a novel integron, In1586. Nine different I1 pMLST types were present, with ST12 (*n* = 13) and ST26 (*n* = 20) being the most represented (Tables 6, 7). Fourteen plasmids could not be typed by pMLST, due to missing alleles. Twenty-one plasmids were isolated from turkey sources and thirteen from chicken (Table 6).

### N Replicons

Four isolates contained N replicon-associated genes leading to four draft plasmids. Three plasmids contained AR genes. IncN pMLST results identified two plasmids that were ST1, one was ST3, and one was untypable. Isolates were four different serotypes and sources (Table 8).

### Q1 Replicons

Q1 replicon associated genes were identified in seven isolates yielding seven draft plasmids containing AR genes. All Q1 plasmids contained AR genes for aminoglycosides and sulfonamides and three also contained *tet*AR genes for resistance to tetracycline. In addition to these five genes, one Q1 plasmid contained an additional aminoglycoside resistance gene, *aph*3-Id. Plasmids were found in isolates of seven different serotypes, and five plasmids were from swine sources (Table 9).

### X Replicons

Thirty-three isolates contained an X1 replicon-associated gene, one contained an X2 replicon-associated gene, and one contained an X4 replicon-associated gene, yielding 29 draft X plasmid sequences (Table 10). The other four isolates with X1 replicons were serotype Dublin, which can contain a virulence plasmid with two replicons, FIIs and X1; therefore, those plasmids were counted as F type (Table 4) (Mohammed et al., 2017). Five of the X1 plasmids and the one X4 plasmid contained *bla*_TEM-1_. The X2 plasmid contained *aph*3″-Ia.

### Co-occurrence

Multiple replicon-associated genes of different types were detected in 92 of 155 isolates containing plasmids (Supplementary Table S1). Incidence of co-occurrence varied by replicon type, but more than half of all plasmids were present with additional replicons in the same isolate. Replicons with the highest frequencies of co-occurrence were X1 (94.2%), HI1 (100%) and HI2 (85%), I1 (75.8%), and Q1 (85.7%) (Table 11). There were three cases of two different replicons present not only in the same isolate but on the same contig, all of which were FIIs replicons with an X1 replicon in *S*. Dublin isolates.

**Table 11:** The co-occurrence of replicons with additional replicons within the same isolate from the retrospective isolates set (*n* = 193).

	AC	F	HI1	HI2	I1	N	Q1	X	pSC101	ColE
AC	32	6	0	5	9	0	0	9	0	14
F	6	43	2	2	7	0	1	7	0	8
HI1	0	2	4	0	2	0	0	2	0	0
HI2	5	2	0	21	9	1	0	2	0	16
I1	9	7	2	9	62	0	1	14	1	34
N	0	0	0	1	0	4	0	0	0	1
Q1	0	1	0	0	1	0	7	1	0	3
X	9	7	2	2	14	0	1	35	0	17
pSC101	0	0	0	0	1	0	0	0	4	2
ColE	14	8	0	16	34	1	3	17	2	76

*Gray boxes indicate the total number of replicons identified*.

### Antibiotic Resistance Cassettes (ARCs)

**FIGURE 1:**
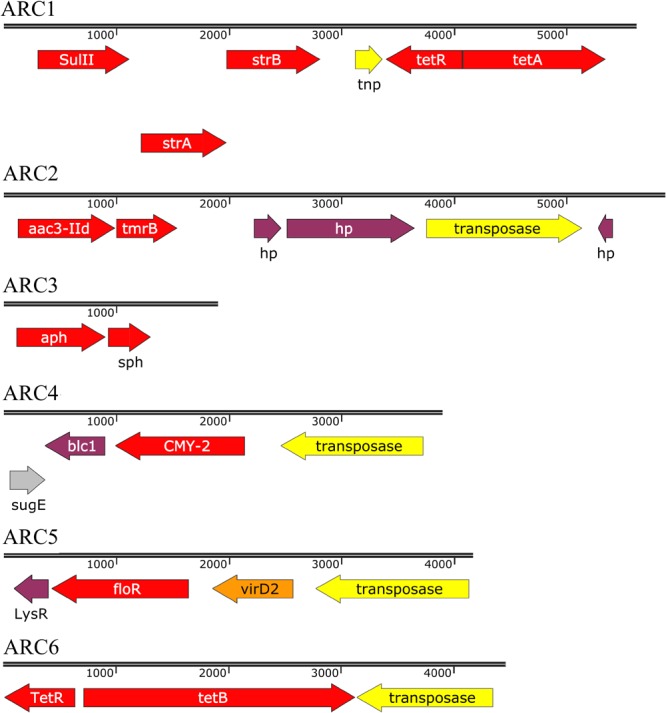
Sequences of antibiotic resistance cassettes (ARCs) identified. Length of sequences are proportional. Arrow color indicates gene classification. Red, AR gene; Yellow, mobile element gene; Gray, metal resistance gene; Orange, relaxase gene; Purple, other gene. Gene abbreviations as follows: tnp, transposase; hp, hypothetical protein.

**FIGURE 2:**
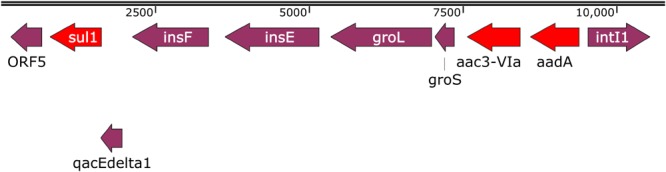
Example sequence of one of the pseudo-ARC variant sequences. Red arrows are AR genes, purple arrows are other genes.

Six ARCs and one pseudo-ARC, as defined in materials and methods, were identified (Figure 1, 2 and Table 12). ARC1 (5627 bp), consisting of *tet*A, *tet*R, *str*A, *str*B, *sul*2, was found in 27 isolates on A/C plasmids and five isolates on Q1 plasmids. ARC2 (5868 bp), consisting of *aac*3-IId and *tmr*B, was present in 11 isolates and located on ColE (1), HI2 (1), and I1 (9) plasmids. ARC3 (1902 bp), consisting of *aph* and *sph*, and was found on eleven HI2 plasmids and two I1 plasmids while ARC4 (3911 bp), containing *bla*_CMY_, *hyp*, and *sug*E, was found on 16 I1 and 28 A/C plasmids. ARC5 (4173 bp), consisting of *flo*R and genes of unknown function, was present on 24 A/C plasmids. ARC6 (4462 bp), containing *tet*B, was located on six F plasmids, 17 HI2, and two HI1 plasmids. ARC6 was also found in two additional isolates but could not be confirmed as associated with a plasmid. The final ARC, designated pseudo-ARC, was an integron (In2 In237, In839, In1581, and In1583), containing *aac*3-Via, *aad*A, and *sul*1 (Figure 2). This ARC was designated pseudo because there was no consensus sequence due to variation in sequence. However, the ARC was still included in the characterization because the genes were identified together on the same contig, all within an integron structure, and in the same order in 22 isolates.

**Table 12:** AR genes contained in each antibiotic resistance cassette (ARC) and their associated replicons from the retrospective isolate set (*n* = 193).

ARC	Associated replicons
ARC1: *tet*AR, *str*AB, *sul*2	A/C(27), Q1(5)
ARC2: *aac*3-IId	ColE(1), I1(9), HI2(1)
ARC3: *aph, sph*	HI2(11), I1(2)
ARC4: *bla*_CMY -2_,	A/C(28), I1(16)
ARC5: *flo*R	A/C(24)
ARC6: *tet*B	F(7), HI1(2), HI2(15)
Pseudo-ARC: *aac*3-Via, *aad*A, *sul*1	A/C(4), HI2(1), I1(16)

*Genes listed are not the only genes contained within the AR ARCs.*

**FIGURE 3:**
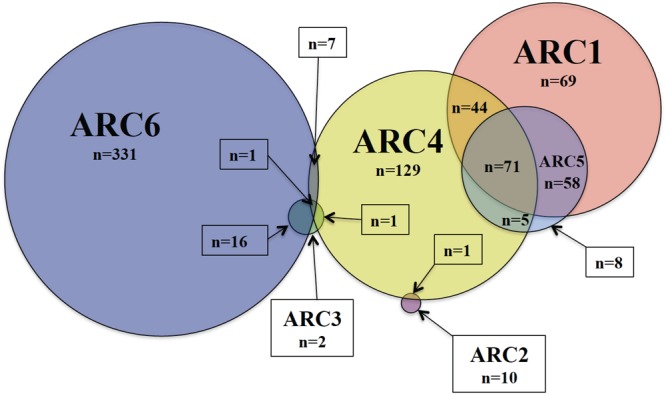
Number of FSIS isolates containing the six ARCs or combination thereof. Total isolates containing each ARC are as follows: ARC1 = 242, ARC2 = 11, ARC3 = 20, ARC4 = 258, ARC5 = 142, ARC6 = 355.

Antibiotic resistance cassettes sequences were identified in *Salmonella*, isolated from 2014 to 2018, sequenced by USDA-FSIS (*n* = 6681) (Figure 3, Table 13, and Supplementary Table S4). ARC1 was found in 242 isolates, 79.8% of which were from cattle. Thirteen different serotypes were represented among the 242 isolates, and the ARC was identified on a contig also containing a plasmid replicon in 43 isolates. ARC2 was found in 11 isolates that were from five serotypes and three different sources. Only one was on a contig with an F plasmid replicon, a serotype Kentucky isolate from chicken. ARC3 was found in 20 isolates. All isolates were serotype Heidelberg except one isolate from swine that was serotype Mbandaka. Two were associated with a plasmid sequence, both HI2 from serotype Heidelberg. ARC4 was found in 259 isolates of 19 different serotypes. Sixty-three were associated with plasmids, types: A/C, F, K, and I1. ARC5 was identified in 142 isolates, of 15 different serotypes, and was associated with a plasmid in 17 isolates. ARC6 was identified in 355 isolates of 23 different serotypes, 78% of which were serotype Kentucky. ARC6 was present on a plasmid in 274 isolates (Table 13). Two hundred and five USDA-FSIS isolates contained multiple ARCs (Figure 3).

**Table 13:** Isolates from FSIS containing the six antibiotic resistance cassette (ARCs) described.

	Total	Animal		
ARC	isolates	source	Serotypes	Plasmid types
1	242	C: 193	Dublin, Heidelberg, Newport, Ohio, Reading, Typhimurium	A/C: 17 I1: 1 Q1: 25
		Ch: 2	Heidelberg, Infantis	
		T: 31	Agona, Heidelberg, I,4,[5],12:i:-, Infantis, Reading, Senftenberg Agona, Derby, Heidelberg, I,4,[5],12:i:-,	
		S: 15	Infantis, London, Muenchen, Ohio, Reading, Typhimurium	
		P: 1	Dublin	
2	11	Ch: 5	Kentucky, Schwarzengrund	F: 1
		T: 5	I,4,[5],12:i:-, London, Schwarzengrund	
		S: 1	Senftenberg	
3	20	C: 1	Heidelberg	HI2: 2
		Ch: 16	Heidelberg	
		T: 1	Heidelberg	
		S: 2	Heidelberg, Mbandaka	
4	259	C: 112	Dublin, Heidelberg, I,4,[5],12:i:-, Newport, Ohio, Reading, Typhimurium	A/C: 16 F: 1 I1: 44 K: 2
		Ch: 113	Cerro, Heidelberg, I,4,[5],12:i:-, Infantis	
		T: 14	Kentucky, Litchfield, Typhimurium Agona, Heidelberg, I,4,[5],12:i:-, Infantis, Litchfield, Liverpool, Montevideo	
		S: 19	Agona, Anatum, Derby, Heidelberg, I,4,[5],12:i:-, Infantis, London	
		E:1	Typhimurium, Uganda, Worthington Typhimurium	
5	142	C: 120	Anatum, Dublin, Meleagridis, Muenster, Newport, Ohio, Reading, Typhimurium	A/C: 16 I1: 1
		Ch: 7	Heidelberg, Infantis, Rough O:r:1,5	
		T: 6	Agona, Heidelberg, Infantis, Senftenberg	
		S: 9	Agona, Derby, I,4,[5],12:i:-, Infantis, Typhimurium	
6	355	C: 11	Anatum, Cerro, Heidelberg, Kentucky, Montevideo	F: 245 I1: 25 HI2: 4
		Ch: 295	8,20:-:z6, Heidelberg, Kentucky, Mbandaka, Oranienburg, Schwarzengrund	
		T: 13	4,[5],12:d:-, 4,[5],12:r:-, Agona, Albany, Berta, 1,4,[5],12:i:-	
		S: 35	Agona, Bovismorbificans, Braenderup, Brandenburg, Derby, Heidelberg, I,4,[5],12:i:-, Infantis, Johannesburg, Kentucky, London, Mbandaka, Uganda	
		RTE: 1	Derby	

*Animal source abbreviations are as follows, C, cattle; Ch, chicken; T, turkey; S, swine; P, unidentified poultry; E, environmental food contact surface; RTE, ready to eat product. The number of isolates containing an ARC associated with a plasmid are total for the isolate set, not per commodity*.

**FIGURE 4:**
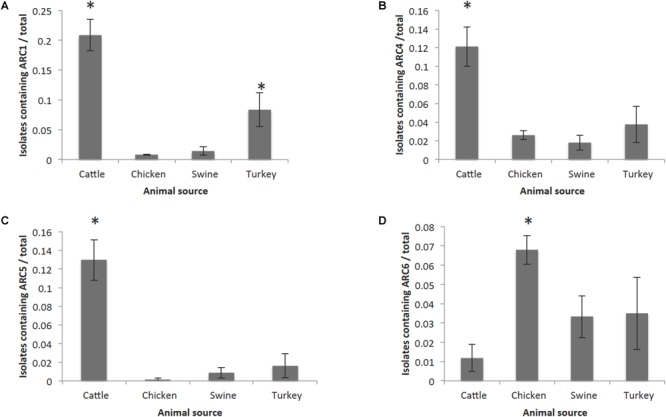
Frequency of animal sources containing each cassette compared to other animal sources. Error bars reflect 95% Confidence intervals (95% CI). Only graphs for ARCs with significant (^∗^) associations are shown. **(A)** Frequency of isolates containing ARC1. **(B)** Frequency of isolates containing ARC4. **(C)** Frequency of isolates containing ARC5. **(D)** Frequency of isolates containing ARC6.

**FIGURE 5:**
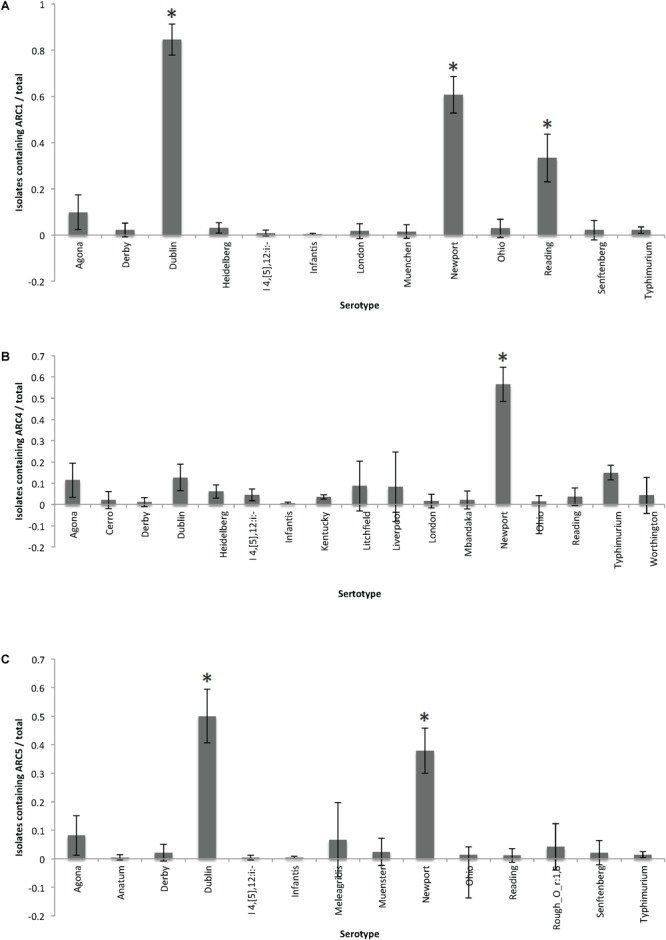
Frequency of serotypes containing each cassette compared to other serotypes for each cassette. Error bars reflect 95% Confidence intervals (95% CI). Only graphs for ARCs with significant (^∗^) associations are shown. **(A)** Frequency of isolates containing ARC1. **(B)** Frequency of isolates containing ARC4. **(C)** Frequency of isolates containing ARC5.

Among these FSIS isolates, animal sources and serotypes were significantly more likely to contain certain ARCs than others. Isolates from cattle sources were significantly more likely to contain ARC1 than any other source (95% CI: 0.18–0.23). Isolates from turkey sources were more likely to contain ARC1 than isolates from chicken and swine (95% CI: 0.06–0.11, Supplementary Data). Isolates from cattle were also significantly more likely to contain ARC4 and ARC5 than any other source (95% CI: 0.1–0.14, 0.12–0.15), while isolates from chicken were significantly more likely to contain ARC6 than other sources (95% CI: 0.06–0.08, Figure 4). Serotype Dublin isolates, which were only identified from cattle sources, and serotype Newport isolates were significantly more likely to contain ARC1 (95% CI: 0.78–0.91, 0.53–0.69) and ARC5 (95% CI: 0.41–0.59, 0.3–0.46) than isolates of other serotypes identified (Supplementary Data). Isolates of serotype Reading were also significantly more likely to contain ARC1 than other serotypes identified, except for Newport and Dublin (95% CI: 0.23–0.44, Supplementary Data). Serotype Newport isolates were also significantly more likely to contain ARC4 than all other serotypes (95% CI: 0.49–0.65, Figure 5).

Antibiotic resistance cassettes were also associated with each other in certain animal sources. Isolates from cattle containing ARC4 had a 90% probability of also containing ARC1, while isolates from chicken only had a 1.8% probability. Isolates from cattle containing ARC5 had a 94% probability of also containing ARC1; however, isolates from cattle that were positive for ARC1 only had a 52 and 58% probability of containing ARC4 and ARC5, respectively. Probabilities of ARC co-occurrence are shown in Supplementary Data.

Antibiotic resistance cassettes sequences were also compared with the NCBI non-redundant database to identify other isolates containing the ARC sequences. ARC1 was found in 88 isolates of 12 different species, 17 types of sources, 14 different countries, and present on A/C, I1, F, HI2, and Q1 plasmids, as well as on the chromosome and on integrative conjugative elements (ICE). ARC2 was identified in 16 different species from 15 countries and in 12 different source types. ARC2 was associated with the highest number of different replicon types including A/C, F, I1, HI1, HI2, L/M, and N. ARC3 was identified in 3 different species, 4 different countries, and from 2 sources, but associated with four different replicon types, F, I1, HI2, and N. ARC4 was identified in 12 different species, 20 different countries, and from 11 sources, but in only three identifiable plasmid types, A/C, I1, and K. ARC5 was identified in 13 different species, 17 different countries, and from 17 sources. Unlike in the retrospective dataset, ARC5 was found in four different replicon types, A/C, F, I1, and HI2, as well as ICEs (*n* = 14). ARC6 was identified in 26 different species, 21 different countries, from 10 sources, associated with four different replicon types F, HI1, HI2, and K (Supplementary Tables S5–S10).

## Discussion

With a goal of investigating the relationship between AR genes and plasmids in *S. enterica* isolates associated with food animals, 193 isolates were sequenced to identify their AR genes and plasmids. The isolates for this retrospective study were selected to represent a great level of diversity, therefore, prevalence of plasmids, ARCs, AR genes, etc. in these retrospective isolates cannot be used to imply their overall prevalence in *Salmonella* associated with animals. Nevertheless, many conclusions can be made with this fact in mind.

More than 80% of AR genes identified were located within a plasmid sequence. The number and diversity of plasmids identified in the set of retrospective isolates indicated that many different plasmids were involved in AR in *Salmonella* among food animals. At least one plasmid of every replicon type identified contained an AR gene. Although certain replicon types were more prevalent than others, no single type was responsible for encoding the majority of the AR genes.

Although *aac6-I* was the most frequently identified gene, these genes are commonly chromosomal genes in *Salmonella* rendered silent by a deletion in the promoter. However, expression can be increased by a fusion of genes upstream (Magnet et al., 1999). No isolates from the retrospective study contained this fusion, despite three isolates showing resistance to gentamicin that lacked any other genes for gentamicin resistance. It is possible that these isolates contain an unknown gene or mutation that confers gentamicin resistance.

A/C plasmids, as a whole, contained more AR genes per plasmid than any other replicon type. Approximately 25% of the total AR genes identified were located on an A/C plasmid despite A/C plasmids only representing 15% of the total number of plasmids identified. Conversely, I1 was the most prevalent replicon type (aside from ColE), accounting for 29% of the total plasmids identified, but only contained 13% of the total AR genes. These findings are consistent with previous studies that isolated A/C and I1 plasmids (Cao et al., 2018). A/C plasmids containing up to 13 AR genes have been identified in isolates from animals in other studies (Hoffmann et al., 2017). I1 plasmids have been seen with similar gene profiles to the profiles detected in this study as well, especially the profile containing the single *bla*_CMY -2_ gene (Folster et al., 2012; Kaldhone et al., 2018). The single pMLST ST2 A/C plasmid found in this study was similar to a previously described ST2 A/C plasmid in that it contained approximately 22,500 base pairs of the *Yersinia pestis* chromosome (Hoffman et al., 2013). These genes from *Y. pestis* encoded a siderophore, methyltransferase, adenylase, as well as other virulence associated functions. The isolate identified in this study was serotype Typhimurium var 5 – from a chicken-associated source, isolated in 2004. It has been recently suggested that IncA/C plasmids are actually two separate incompatibility groups: IncA and IncC (Ambrose et al., 2018). By that classification, all A/C plasmids from the retrospective study would be considered IncC.

Interestingly, many A/C containing isolates also harbored an additional replicon, which could increase the transferability of AR genes from these isolates to others (Han et al., 2018). A/C plasmids occurred with additional replicons 23/32 times and did not occur with HI2 plasmids unless an I1 and a ColE replicon was also present. Those five isolates were the only isolates to have more than two large plasmids in the same isolate. All five of those isolates were from a turkey source and four were of serotype Heidelberg with the fifth being serotype Bredeney. Fourteen of the 23 isolates contained both an A/C and an additional plasmid of a different replicon. The additional plasmid contained AR genes different and in addition to those on the A/C plasmid. As suggested in Han et al. (2018), carriage of multiple plasmids may positively affect transfer of AR genes. It may also affect the transferability of A/C plasmids, including those without the genes required for transfer. While the study by Han et al. (2018) was only conducted in A/C positive isolates, it is possible this effect is present among isolates containing other combinations of replicons.

Although F type plasmids had one of the lower percentages of plasmids containing AR genes, these are of particular interest because several virulence plasmids belong to the F incompatibility group. Certain *Salmonella* serotypes, like Typhimurium and Enteritidis, usually contain an F replicon characterized by the *spv* genes for enhanced virulence as seen in the pSLT plasmid of *S. enterica* serovar Typhimurium strain LT2 (Boyd and Hartl, 1998; Silva et al., 2017). Of the 14 F plasmids identified with AR genes, four of those are variants of *Salmonella* virulence plasmids. In five isolates containing F-type plasmids, the plasmid was a variant of an avian pathogenic *E. coli* (APEC) plasmid that has been seen previously in *Salmonella* serotype Kentucky (Fricke et al., 2009; Johnson et al., 2010). Predictably, these five isolates were also serotype Kentucky and came from poultry sources. Additionally, one plasmid appears to be similar to a virulence plasmid of the fish pathogen, *Edwardsiella tarda* (Yu et al., 2012).

All HI type plasmids identified contained AR genes. HI1 and HI2 plasmids both contained *tet*B associated with ARC6, which is a portion of Tn10. This is also consistent with previous findings indicating an association between Tn10 and HI type plasmids (Cain and Hall, 2012a,b). However, HI2 plasmids identified in this study were largely untypable by pMLST despite containing every gene used in the scheme, due to a mutation in one of the alleles. This predicts that these plasmids belong to a new sequence type and may indicate a new lineage of HI2 plasmids, different from the sequenced plasmids used to develop the pMLST scheme (Garcia-Fernandez and Carattoli, 2010).

The seven Q1 plasmids identified were consistent with previously reported plasmids with the exception of additional AR genes found on the Q1 plasmids in this study. Q1 plasmids generally have a well-conserved structure with the differences being confined primarily to the AR genes (Loftie-Eaton and Rawlings, 2012). Five of the plasmids contained *tet*AR genes for tetracycline resistance, which are rare in Q1 plasmids, but have been seen in Europe and the United States (Oliva et al., 2017). The plasmids isolated were mostly from swine sources, but were also found in ground beef as well as one unknown source. Five of the Q1 plasmids isolated contained ARC1, which was also present on A/C plasmids. Interestingly, only three Q1 plasmids co-occurred in isolates with potentially conjugative plasmids. Since Q1 plasmids cannot transfer unless another conjugative plasmid is present, this likely indicates that four of the seven Q1 plasmids would be unable to transfer to other bacteria without the acquisition of a conjugative plasmid (Frey et al., 1992).

With the exception of ARC5 which was found only on IncA/C plasmids, all ARCs were present on multiple replicon types, indicating that the prevalence of these ARCs is not due to the expansion of a single clonal plasmid. In the NCBI databases, ARC5 was associated with multiple replicon types and therefore cannot be considered exclusive to the A/C replicon. In the retrospective isolate set, every plasmid-associated *flo*R gene was a part of ARC5. Two additional isolates contained the *flo*R gene but as part of *Salmonella* Genomic Island One (SGI-1) which did not share the ARC structure. ARC1 was the only ARC not associated with a transposase gene, possibly indicating that the MGE structure originally associated with ARC1 has been lost or that the MGE was lost in assembly.

In contrast to the retrospective isolates, the isolates collected by USDA-FSIS can be used to predict the frequency of the ARCs in the *Salmonella* population found currently among food animals over the past 4 years. More than 75% of the isolates containing ARC1 and more than 80% of isolates containing ARC5 were isolated from cattle associated sources. However, only around 40% of the isolates containing ARC4 were associated with cattle despite many of the isolates containing both ARC1 and ARC4 or all three ARCs. A higher percentage of chicken-associated isolates containing ARC4 was responsible for that reduction in percentage, with 37% of ARC4 isolates coming from chicken-associated sources as compared to 2% and almost 4% for ARC 1 and ARC5.

Cattle isolates from USDA-FSIS had a significantly higher chance of containing ARC1, ARC4, and ARC5 than all other sources. This is to be expected, as these three ARCs were associated with A/C plasmids when identified together in the retrospective isolate set. In the USDA-FSIS samples, 12 isolates had ARC1, ARC4, and ARC5 associated with an A/C plasmid. A/C plasmids carrying multiple AR genes have been frequently shown to be associated with isolates from cattle (Carattoli, 2009; Lindsey et al., 2009).

Chicken sources, however, had a significantly higher chance of containing ARC6 than other sources. This could be due to an association with serotype Kentucky, which was the most commonly isolated serotype from the FSIS isolate set. While not significantly more likely to contain the ARC than all the other serotypes, serotype Kentucky did have the third highest frequency of isolates containing the ARC, but was also the most frequently isolated serotype in the isolate set. *Salmonella* Kentucky isolates containing an APEC colV plasmid have been identified that contain ARC6 on that plasmid (Fricke et al., 2009; Johnson et al., 2010).

ARC2 and ARC3 were both detected infrequently in the FSIS isolate set. ARC2 was not found in any isolates from cattle but the 11 isolates were from five different serotypes. In contrast, the 20 isolates containing ARC3 were only comprised of two serotypes, Heidelberg and Mbandaka. Similarly, the majority of isolates in the retrospective isolate set that contained ARC3 were serotype Heidelberg.

The plasmids associated with each ARC in the FSIS sequences were also consistent with those identified in the retrospective isolate set; however, additional plasmid replicon types were associated with the ARCs. ARC1 was associated with A/C and Q1 as in the retrospective isolate set, but was also associated with one I1 plasmid. ARC4 was found on A/C, I1, K, and F plasmids, whereas ARC4 was seen only on A/C and I1 in the retrospective isolates. While only a fraction of the identified ARCs could be associated with a plasmid sequence, this does not mean that the ARCs identified in other isolates were not associated with plasmids. Further characterization of those isolates including assembly of plasmid sequences would be necessary to determine the location of all ARCs. However, the ARCs that were associated with plasmids indicated similarity between the retrospective isolates and the isolates recently collected by FSIS. Whether serotype or source is the correlating factor for plasmids identified cannot be determined without further investigation.

Every ARC identified in this study was also found in other bacteria when compared to the NCBI NR database. While the species represented are limited by what has been sequenced by others, the presence of the ARCs in these organisms indicates that these ARCs are not limited to *Salmonella* and have the ability to persist and confer AR to a diverse group of bacteria belonging to at least two orders, enterobacteriales and vibrionales. ARC1, ARC4, and ARC5 in particular were identified in A/C plasmids from *E. coli* isolates in a 2011 study by Fernandez-Alarcon et al. (2011). This study also suggested that in A/C plasmids, ARC1 and ARC5 may be adjacent.

In contrast to the retrospective isolate set, some of the ARCs in isolates from NCBI were not plasmid associated, but instead associated with ICEs or incorporated into the chromosome. ARCs were also present in other isolates with varying frequencies. ARC4, ARC5, and ARC6 were found in over 100 isolates, while ARC2 was found in less than 10. While this is similar to what was identified in both the retrospective and FSIS isolates, this may reflect sequencing bias rather than infrequent presence of ARC2 and ARC3.

Overall, the plasmids identified in this study showed diversity, but also showed similarities among replicon types. While the plasmids shared homologous sequence with previously sequenced plasmids, there were also novel sequences. Additional investigation is needed into individual plasmids to further characterize each replicon type. It still remains to be determined why some AR genes were found on some replicon types, but not others, as well as if the plasmids that did not contain AR genes harbored other genes beneficial to the host bacterium. Answering these questions will further advance the knowledge of how AR genes are spreading in *Salmonella* as well as in agricultural environments.

## Author Contributions

EM, LW, CJ, JW, MS, GT, MM, and JF contributed to the conception and design of the experiments. EM, SG, TJ, LH, TW, JB, JW, and MS contributed to the generation and analysis of data. EM wrote the manuscript. All authors contributed to the revision of the manuscript and approved the submission.

## Conflict of Interest Statement

The authors declare that the research was conducted in the absence of any commercial or financial relationships that could be construed as a potential conflict of interest.

## Footnotes

1 https://www.ncbi.nlm.nih.gov/pathogens/isolates#/search/

2 https://www.cdc.gov/narms/antibiotics-tested.html

3 http://cab.spbu.ru/software/spades/

4 https://pubmlst.org/plasmid/

5 http://www.snapgene.com/
