# Supplementary material for: Antimicrobial Resistance Genes, Cassettes, and Plasmids Present in Salmonella enterica Associated With United States Food Animals
Source: Front Microbiol. 2019 Apr 17;10:832. doi: 10.3389/fmicb.2019.00832 (PMC6479191; doi:10.3389/fmicb.2019.00832)
Supplement: Supplementary file 3 [file Table_3.docx]

Supplementary Material

Antimicrobial Resistance Genes, Cassettes, and Plasmids Present
in *Salmonella enterica* Associated With United States Food Animals

Elizabeth A. McMillan, Sushim K. Gupta, Laura E. Williams, Thomas Jové, Lari M. Hiott, Tiffanie Woodley, John B. Barrett, Charlene R. Jackson, Jamie Wasilenko, Mustafa Simmons, Glenn E. Tillman, Michael McClelland, Jonathan G. Frye^*^

*** Correspondence:** Jonathan G. Frye, jonathan.frye@ars.usda.gov

**
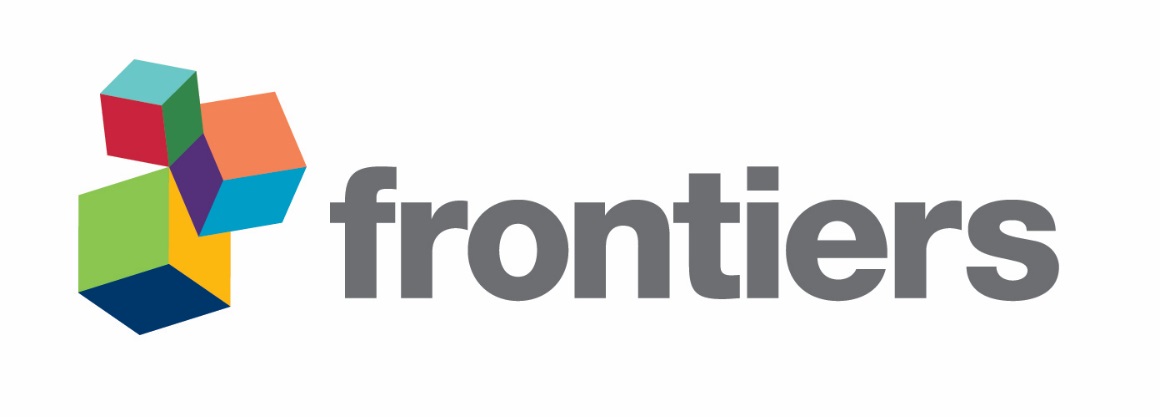
**

**Supplementary Table 1.** Metadata, genotypic, and phenotypic information for each isolate in the retrospective isolate set. Blank cells in the year, source, and source category columns indicate unknown information. Blank cells in all other columns indicate that the isolate does not contain the replicon, gene, or ARC. For phenotypic data, R indicates resistant, blank indicates susceptible or intermediate. For colE, rep = replion associated gene, rel = relaxase gene.

**Supplementary Table 2.** Assembly statistics for 193 retrospective isolates.

**Supplementary Table 3.** Isolates containing ColE plasmids with AR genes on the same contig as the replicon gene. *Anatum variant

**Supplementary Table 4.** Metadata and AR cassette profile of FSIS isolates positive for at least one cassette. Additional metatdata is available from NCBI Pathogen Browser. Empty cells in the cassette columns indicate that the isolate does not contain that cassette. Colored cells coorespond to plasmid types listed in the key and indicate that the cassette is located on a contig containing a plasmid replicon in that isolate.

**Supplementary Table 5.** Isolates from NCBI containing ARC1.

**Supplementary Table 6.** Isolates from NCBI containing ARC2.

**Supplementary Table 7.** Isolates from NCBI containing ARC3.

**Supplementary Table 8.** Isolates from NCBI containing ARC4.

**Supplementary Table 9.** Isolates from NCBI containing ARC5.

**Supplementary Table 10.** Isolates from NCBI containing ARC6.

**Supplementary Data.** Includes data and calculations for cassette, animal source, and serotype confidence intervals, and conditional probabilities.
